# Developing entrustable professional activities for doctoral graduates in health professions education: obtaining a national consensus in Iran

**DOI:** 10.1186/s12909-022-03469-6

**Published:** 2022-06-02

**Authors:** Reza Zaeri, Roghayeh Gandomkar

**Affiliations:** 1 Department of Medical Education, School of Medicine, Tehran University of Medical Sciences, No. 57, Hojjatdust Alley, Naderi St., Keshavarz Blvd, Tehran, 141663591 Iran; 2 Health Professions Education Research Center, Tehran University of Medical Sciences, Tehran, Iran

**Keywords:** Entrustable professional activities, Health professions education, Doctoral program

## Abstract

**Background:**

The number of doctoral programs in health professions education (HPE) is expanding. Entrustable professional activities (EPAs) can be a mechanism to define the expected activities of the HPE doctorate to inform training and assessment processes. The purpose of this study was to develop and reach a consensus on EPAs for HPE doctoral graduates.

**Methods:**

We used a modified Nominal Group Technique (NGT) to elicit EPA titles followed by two rounds of a modified Delphi survey to seek consensus on the EPAs among groups of experts (HPE doctoral graduates and Board of HPE Examiners members) at the national level in Iran between July 2019 and July 2020.

**Results:**

A total number of 92 initial EPA titles, which emerged from brainstorming in the NGT meeting, was reduced to 27 titles during the clarification process. The final EPA framework consisted of 24 EPA titles with descriptions, arranged in three categories: Research and scholarship (6 EPAs), Educational development (11 EPAs) and Educational management (7 EPAs). All final EPAs scored ≥80% agreement at the national level.

**Conclusions:**

The proposed EPAs framework can be used to improve the HPE doctorate training and to inform employment decisions. A future international consensus procedure could use these EPA outcomes as a starting point.

**Supplementary Information:**

The online version contains supplementary material available at 10.1186/s12909-022-03469-6.

## Background

The number of doctoral degree-awarding programs in health professions education (HPE) is progressively expanding [1]. Many academic institutions, HPE scholarship units, professional associations and health care delivery centers hire graduates with such advanced degrees to support innovations in curriculum, optimizations in teaching and learning, implementation of programmatic assessment and initiatives in quality assurance as well as engagement in scholarship activities, resulting in publication [2]. While there may be a general understanding of what these scholars can be employed for, activities that can be expected from doctoral graduates in HPE have not been defined.

Doctoral programs in HPE generally adopted the traditional North American model which requires coursework, research, publications and a dissertation. In several other programs, students could acquire a Doctor of Philosophy (PhD) by choosing to specialize in HPE under the broader programs of higher education, psychology or sociology. There are also some programs that did not follow a structured curriculum, but candidates complete several publishable research projects through supervision and apprenticeships [3]. The most important goals of the doctoral programs in HPE are to prepare students to conduct robust research, − pursue a teaching career at academic institutions, − identify complex problems in educational systems and produce innovative solutions aimed to improve the quality of life at individual, organizations and society levels [4–7].

The literature about HPE doctoral programs is mainly focused on listing the institutions that offer formal or less structured doctoral programs [1] and providing basic information about the role of programs and their various options [8], organizational structure [9], the process of supervision and quality of research [10]. Other studies provided standards for PhD dissertations in HPE [11] or proposed tips for studying a PhD in HPE [5, 12]. One paper compared the instructional content of Master’s and PhD programs in HPE and mapped the links between the content of HPE programs and continuous professional development (CPD) roles and responsibilities [13]. Despite the growing desire for recruitment of HPE doctoral graduates, the published literature does not necessarily address the expected activities that graduates in the HPE doctorate should be ready to perform.

The Entrustable Professional Activities (EPAs) framework may be a mechanism to define the activities the HPE doctorate may be expected to do. The traditional categorization of educational goals into cognitive (knowledge), psychomotor (skills) and affective (attitude) domains of educational objectives have been criticized for being somewhat prescriptive and for the lack of authenticity. Competencies were used to address the challenge by integrating knowledge, skills and attitude. However, both educational objectives and competencies are attributes of individuals and are invisible until they are being used while the individual is performing tasks or activities [14]. On the other hand, EPAs describe activities of a profession that are performed by qualified persons, independently and proficiently; requiring integrated competencies in different areas in order to perform activities effectively [15]. The description of EPAs can guide doctoral supervisors as well as trainees concerning the extent, specificity and context of the training and assessment and inform administrators, graduates and future employers about the expected activities [16].

There is a large body of literature on the development and implementation of EPAs in health care education [17] including residency [18, 19] and undergraduate medical education programs [20]. The EPAs of these medical training programs are mainly concentrated on different aspects of practice in a clinical environment while activities related to doctoral programs are concerned with the requirements for an academic environment such as research and teaching. There is also a growing number of studies describing EPAs for non-clinical aspects of medical education such as research and scholarship practice (translational scientists [21]), managerial activities (simulation leaders [22] and program directors [23]) and teaching qualification for university teachers in the health professions [24, 25], in the context of faculty development for basic teaching activities [26] and small group facilitation [27, 28]. In these studies, EPAs were defined as closely as possible to the original concept originating from the clinical workplace. Authors used consensus methods such as (modified) Delphi method or Nominal Group Technique (NGT) to develop titles and descriptions of EPAs. No studies defined full descriptions of EPAs [16]. Dewey et al. (2017) proposed a full description of one EPA for university teachers in the health professions as an example in their paper (Personal View) [24]. We found only one study related to EPAs in HPE scholarship. University of Michigan Medical School developed a set of 20 EPA titles for a competency-based Master’s program in HPE and incorporated it into all learning experiences [29] and the assessment procedures for the Master candidates [30]. We did not find similar approaches in HPE doctoral programs. Given the lack of studies in doctoral training and the distinctive features of this program, the purpose of this study is to develop and reach a consensus on EPAs for doctoral graduates in HPE in Iran.

The first doctoral program started in 2010 in Iran. Ten years later, five Departments for HPE offered this degree around the country. These doctoral programs follow the North American model and the goals of the programs have been defined (the same for all five departments) as designing and conducting rigorous research projects in HPE, promoting innovations and development projects and supporting the process of change. In 2015, a national project was started to develop the competency framework for doctoral graduates in HPE to guide HPE doctoral curriculum [31]. The proposed EPA framework could be applied in other HPE doctoral programs with similar aims and structure.

## Methods

### Setting and participants

This study was conducted in the context of doctorate programs in HPE in Iran between July 2019 and July 2020. Participants in our study were doctoral graduates of HPE and members of the Board of HPE Examiners. There were 51 HPE doctoral graduates at the time of this study. Eleven doctoral graduates in HPE were invited for the first phase of the study via email using purposive sampling. The graduates were selected based on their continued activity in the field of HPE after graduation. We considered maximum variation and deliberately recruited graduates from different work settings (HPE departments, HPE research centers, HPE developmental centers) to ensure variation in ideas. For the second phase, we used the census method and all eleven members of the Board of HPE Examiners and all HPE doctoral graduates who were not invited for the first phase (*n* = 40) were requested to complete an online questionnaire.

### Procedure

The study was performed in two major phases: modified NGT and modified Delphi study.

#### Phase 1: nominal group technique

We used a modified NGT to elicit EPA titles (a general item in a job description and not as specific for a person or a context) of doctoral graduates in HPE in July 2019. The NGT facilitates the participation of all group members to generate fresh ideas through a brainstorming format [32]. Since the concept of EPAs is new to the training of doctorate in HPE, we held an NGT session to develop an initial list of EPA titles to include in a Delphi process.

The meeting was led by three moderators, two of whom were the authors of this paper. The third moderator was the previous head of the Department of HPE at a university with the experience of teaching HPE doctoral students. The meeting lasted four hours. The first phase of the NGT meeting started with providing background information; a brief description of the NGT process (i.e. silent idea generation, presenting and recording ideas, and clarifying and prioritizing ideas) followed by an introduction to the necessity of defining EPAs, their definition and its difference with competencies. Competencies are descriptors of individual graduates while EPAs are descriptors of work. EPAs usually entail multiple competencies in an integrative way and they are a means to translate competencies into the workplace [33]. This was followed by an open conversation to raise questions and concerns about the EPAs and project. Two questions were then projected on a screen: “What do doctoral graduates in HPE do after graduation?” and “Can these activities be captured in EPAs?” Participants were then asked to write down their proposed EPAs in silence (silent idea generation). In the third phase, each participant shared one EPA from their list with the group in a round-robin format. Participants took turns, not allowed to name or react to any previously mentioned items. This cycle continued until no new ideas emerged. All mentioned EPAs were directly typed by one author (RZ) and projected on the screen, allowing the whole group to read a growing list of EPAs. After that, with the assistance of the moderators, participants discussed the list of EPAs that were not clear to them and made clarifications. Appreciating the participants’ limited time, other steps were performed off-site.

After meeting, the authors of this paper reviewed the initial list of EPAs, combined similar EPAs and afterward grouped related EPAs into categories and selected a label for each category. We sent the EPA titles to the NGT participants digitally and asked them to provide their qualitative comments and suggest more data combinations if needed. This clarification phase with participants was repeated twice. After the final refinement, we asked participants to rank the EPAs on a Likert scale, being 1 “not very important” for doctoral graduates in HPE and 5 “very important”. Voting results were then summed across participants.

#### Phase 2: modified Delphi study

We performed two rounds of a modified Delphi survey to seek consensus on the EPA titles among wider groups of experts at the national level between December 2019 and July 2020. The Delphi technique uses multiple rounds to reach an agreement on specific items among geographically dispersed participants [32]. In Delphi round 1, EPA titles obtained from NGT were sent to the study panels digitally. Participants were asked to (1) rate the “importance” of each EPA based on a Likert scale from 1 to 5, where 1 was “not very important” and 5 was “very important”, (2) check for modifications or changes needed for each EPA and (3) identify ignored titles of EPAs. The authors participated in one meeting of the Board and explained the aim and method of the study and invited members to participate. Next, participants allowed to raise concerns and questions and receive clarifications. Graduates were invited via email, confirmed by personal contact.

EPA titles were modified based on the quantitative and qualitative results of the first Delphi round. All qualitative comments were reviewed by the authors, which led to major revisions in the general structure and wording of the titles. A new title was added to the list if two participants mentioned it. Modifications were made even if EPAs scored ≥80% agreement if suggested by at least 2 members of the panel or after a consensus discussion between the researchers reviewing the comments. By reviewing the comments, the authors concluded that developing the EPA descriptions would make them more clear. Therefore, in the next step, one author (RZ) elaborated on the EPA descriptions for each of the EPA titles based on the recommended “features” provided by ten Cate & Taylor (2020) [16]. Descriptions of EPAs were discussed in a panel of 4 specialists in HPE. Finally, another author (RG), with experience in HPE training and research, reviewed and edited the descriptions. The elaboration process led to further combining EPA titles with overlapping content.

During the Delphi round 2, panel members were provided EPAs descriptions including title, specifications and limitations and potential risks in case of failure. Experts were told the mean responses from the first round. Because we had made major revisions in EPA titles, we supplied all EPAs in the second phase. Participants were asked to rate the “importance” of each EPA for doctoral graduates in HPE based on a Likert scale from 1 to 5, where 1 was “not very important” and 5 was “very important”. There was a box for each EPA title and descriptions for comments. There was no need for a third Delphi round since all EPAs fulfilled the agreement criteria in the second round. Refinement was made in EPA titles and descriptions based on the qualitative comments.

### Data analysis

Descriptive statistics were calculated using IBM SPSS statistics 23 including frequency, mean (M) and standard deviation (SD). We defined consensus as ≥80% agreement for a rating of 4 (moderately important) and 5 (absolutely important).

## Results

Nine of eleven doctoral graduates in HPE agreed to participate in the NGT meeting. There were 34 (response rate = 0.85) and 26 (response rate = 0.65) doctoral graduates in HPE who completed the survey in Delphi round 1 and 2, respectively. Nine (response rate = 0.82) members of the Board of Health Professions Education Examiners participated in two rounds of Delphi. In all, 73% (38 out of 52) of study participants were female. The largest number of participants 83% (41 out of 52) worked in HPE scholarship units.

### Phase 1: nominal group technique

A total number of 92 initial EPA titles emerged from brainstorming and clarification in the NGT meeting and was reduced to 75 titles in five areas of consultation, research and scholarship, education, management and evaluation during combination by the research team and then to 27 titles during the iterative clarification process by the NGT participants. All 27 EPA titles scored 100% agreement throughout the voting phase. The number of EPAs in each domain were: Consultation (*n* = 6), Research and scholarship (*n* = 5), Education (*n* = 7), Management (*n* = 5) and Evaluation (*n* = 4).

### Phase 2: modified Delphi study

The titles of EPAs, levels of agreement as well as means and standard deviations per EPA of the first Delphi round are demonstrated in Supplemental Table 1. Out of all EPAs, 16 scored ≥80% agreements with 10 showing 100% agreements. The remaining 11 EPAs scored slightly lower than the threshold (78% agreement). Based on the qualitative comments, the general structure of the EPA framework was adapted and the EPAs were rearranged into three categories: Research and scholarship (6 EPAs), Educational development (11 EPAs) and Educational management (7 EPAs). Table 1 provides a map of the changes made in the general structure of the EPAs. Overall, 4 EPAs were deleted, 5 were merged (3 combined and shaped one new EPAs and 2 others each merged into one existing EPA), one split into 2 EPAs, 4 new EPAs emerged and as a result, 24 EPAs developed. Deleted EPAs demonstrated good agreement in terms of importance yet based on the comments they were nested in other EPAs and seemed redundant. The research and scholarship domain showed the least changes.

**Table 1:** Changes in general structure of EPAs based on the results of the first Delphi round

EPAs		New structure	
		EPA number	Domain
**Domain 1: Consultation**	EPA 1	EPA 6	Research & scholarship
	EPA 2	Merged, EPA 17	Educational development
	EPA 3	Merged, EPA 17	–
	EPA 4	Merged, EPA 17	–
	EPA 5	EPA 24	Educational management
	EPA 6	EPA 12	Educational development
**Domain 2: Research & scholarship**	EPA 7	EPA 1	Research & scholarship
	EPA 8	Merged, EPA 1	–
	EPA 9	EPA 2, EPA 3	Research & scholarship (2)
	EPA 10	EPA 4	Research & scholarship
	EPA 11	EPA 5	Research & scholarship
**Domain 3: Education**	EPA 12	EPA 11	Educational development
	EPA 13	EPA 9	Educational development
	EPA 14	Merged, EPA 8	–
	EPA 15	Deleted	–
	EPA 16	EPA 8	Educational development
	EPA 17	EPA 20	Educational management
	EPA 18	EPA 10	Educational development
**Domain 4: Management**	EPA 19	Deleted	–
	EPA 20	EPA 18	Educational management
	EPA 21	Deleted	–
	EPA 22	Deleted	–
	EPA 23	EPA 23	Educational management
**Domain 5: Evaluation**	EPA 24	EPA 14	Educational development
	EPA 25	EPA 16	Educational development
	EPA 26	EPA 15	Educational development
	EPA 27	EPA 13	Educational development
	New	EPA 7	Educational development
	New	EPA 19	Educational management
	New	EPA 21	Educational management
	New	EPA 22	Educational management

Table 2 demonstrates the finalized titles of EPAs, levels of agreement, means and standard deviations for 24 EPAs rated during the second Delphi round. All EPAs scored ≥80% agreement, 7 with full agreement. The lowest consensus was on EPA 22 and 23 in the educational management domain. Revisions were made in EPA titles (minor refinement) and descriptions (major modifications) based on the qualitative comments. The final EPA descriptions (title, specifications and limitations and potential risks in case of failure) can be found in Supplemental Table 2.

**Table 2:** Levels of agreement, means and standard deviations per EPA of the second Delphi round

EPAs		EPA title	EPA importance	
			Level of agreement (%)	Mean (SD)
**Domain 1: Research & scholarship**	EPA 1	Identifying and translating educational needs to research	97	4.80 (0.47)
	EPA 2	Conducting and analyzing research	97	4.77 (0.42)
	EPA 3	Collaborating, directing and supervising research teams	100	4.71 (0.56)
	EPA 4	Writing, publishing and communicating scientific reports	100	4.60 (0.49)
	EPA 5	Reviewing research and scholarship activities	97	4.51 (0.60)
	EPA 6	Consulting on research and scholarship in HPE	97	4.49 (0.65)
**Domain 2: Educational Development**	EPA 7	Designing and conducting educational needs assessment	97	4.54 (0.60)
	EPA 8	Developing, implementing and revising curricula and educational programs	100	4.86 (0.35)
	EPA 9	Instructional designing for various teaching and learning situations	94	4.83 (0.45)
	EPA 10	Designing and producing educational content in HPE	100	4.69 (0.57)
	EPA 11	Teaching and facilitating in various educational situations	94	4.66 (0.47)
	EPA 12	Mentoring stakeholder groups in HPE	91	4.71 (0.51)
	EPA 13	Reviewing educational materials and products	94	4.60 (0.55)
	EPA 14	Designing, implementing and revising student assessment system	100	4.54 (0.50)
	EPA 15	Designing, implementing and revising the faculty evaluation system	97	4.54 (0.50)
	EPA 16	Designing, implementing and revising quality assurance system	94	4.63 (0.54)
	EPA 17	Consulting on planning, teaching and learning processes, and evaluation activities	100	4.66 (0.53)
**Domain 3: Educational Management**	EPA 18	Analyzing, formulating and revising educational policies	97	4.80 (0.47)
	EPA 19	Designing, implementing and evaluating reforms	100	4.71 (0.45)
	EPA 20	Designing, implementing and evaluating personal and professional support and development programs	91	4.63 (0.64)
	EPA 21	Managing organizational processes and resources	91	4.49 (0.65)
	EPA 22	Managing and supervising projects	89	4.31 (0.67)
	EPA 23	Analyzing the cost-effectiveness of practices and interventions	89	4.43 (0.77)
	EPA 24	Consulting on management and leadership	94	4.57 (0.60)

*HPE* Health Profession Education, *BEME* Best Evidence Medical Education

## Discussion

To our knowledge, this is the first study to propose an EPA framework for doctoral graduates in HPE. Using NGT followed by modified Delphi methodology with doctoral graduates in HPE and members of the Board of HPE Examiners, this study led to a national consensus on 24 EPAs, categorized into research and scholarship (6 EPAs), educational development (11 EPAs) and educational management (7 EPAs). The number of EPAs in our proposed framework resembles the study on EPAs (20 title statements) for the Master’s program in HPE at the University of Michigan Medical School [29]. The recent report on EPAs in all Dutch specialty programs aligns with this notion as well [34]. The overlaps between our framework and EPAs proposed for the Master’s program in HPE [29] may support common activities for HPE scholars. More specifically, the EPAs’ dimensions of this study are in line with the area of activities (i.e. research, teaching and educational development) in that doctorate-trained HPE scholars are often involved in HPE scholarship units [35, 36]. Our participants were mostly the staff of HPE scholarship units and as expected they mentioned a range of activities for HPE professionals.

EPA statements related to the domain of research and scholarship had the highest level of agreement among participants in terms of importance. This domain underwent the least revisions during the consensus process. These findings are not surprising since research and scholarship activities serve as a core task during doctorate programs and it serves as a starting point of discussion among professionals [37]. Many doctorate graduates are recruited in HPE scholarship units as research scientists and are engaged in a range of scholarly activities such as those we obtained in the research and scholarship domain. Etmanski et al. (2020) showed that all HPE research scientists working in HPE scholarship units throughout Canada emphasized their career success in terms of a research-intensive pursuit resulting in peer-reviewed, evidence-based research manuscripts [38]. Similarly, in Iran, involving in research projects and publishing the results in peer-reviewed journals is one of the requirements for faculty promotion [39].

In the educational development domain, EPAs are associated with innovation and improvement in educational programs mainly by collaborating, advising and consulting on curriculum development or revision, instructional design, teaching and facilitation, resource material development, and design or redesign of student assessment and quality assurance system. A large number of EPAs proposed for this domain may be explained by the fact that many innovation projects are running at medical schools and other HPE settings (under the direction of HPE scholarship units) around the country [40] and this requires doctorate graduate involvement in the projects. In line with this explanation, Kahlke and Varpio (2019) demonstrated that HPE scholarship units’ works were defined based on two dominant logics: research and service in the Canadian HPE context [41]. They showed that research or service activities were made important through their association with institutional orders and the context in which they were employed. The next step for our study would be the refinement and validation of EPAs based on the task analysis of HPE doctorate graduates in their work setting. We also recommend exploring the perceptions of experts and HPE doctorate graduates from other settings on the framework.

Although all the EPA statements developed in this study met the criteria for consensus, there was less agreement on several EPAs in the category of educational management and this domain underwent the most changes throughout the consensus phases. The reason may be that our respondents had experienced different job positions during their careers in terms of involvement in administrative and leadership tasks and had different perceptions of educational management activities. If we asked participants to identify essential or desirable EPAs, we might observe more consensus for this question through this domain. Further research should be conducted to make distinctions between EPAs that are required (core) or desirable (elective) for doctorates in HPE to do without supervision [42]. The emphasis on certain EPAs and the required level of unsupervised practice toward the end of training may differ between training programs and it can be another inquiry line.

The methodology of the present study included a sequential multi-step approach of drafting preliminary EPAs and revising these EPAs via a modified Delphi approach that resembles previously published studies on developing EPAs. We incorporated the doctorate graduates’ inputs in the processes of development in addition to experts’ opinions (board members) which results in EPAs reflecting the practice patterns of graduates. It is important particularly since the job description of HPE doctorate graduates is less studied. Another advantage of our study was that we devoted much time and effort to obtain qualitative comments from participants and discussions with experts to enrich our data which is necessary for the HPE as an emerging field.

## Implications

The developed EPA framework, titles with detailed descriptions for each EPA, has several implications in practice. First, it offers a way to translate competencies [31] to professional practice as they describe the activities that a competent HPE professional should be trusted to do. The next step for us would be to map the identified EPAs into relevant competency domains. Second, identified EPAs can be used to help program directors implement structured training grounded in professional activities and to guide quality assurance. Additionally, it can be used as a tool for observing (direct observation of performance or indirect observations of evidence of achievement) the individual learning processes and for providing meaningful feedback based on these observations.

The framework may also be used as a basis for making entrustment decisions and be ensured that doctoral trainees acquired competencies. Almost all experiences regarding the use of EPAs for entrustment decisions are related to medical education programs where patient safety is an ultimate goal and this may supply absolute standards of competence that cannot be compromised. However, graduate degrees in education such as the HPE PhD are often much less standardized and mirror deep personal development in a path that usually differs from others. Supervision of the PhD activities may also be somewhat different than for patient care activities. These variations make the entrustment decisions a complex endeavor in the context of a competence-based HPE program. The case study of the EPAs assessment by the University of Michigan Medical School highlighted that the goals and format of the HPE Master’s program would result in varied and individualized approaches to each EPA by the learners which necessitate a novel and flexible assessment approach [30]. Although EPAs were originally used in clinical practice that was featured by entrustment decisions, recent standardization of teaching practices and research procedures allows EPAs to be proposed for academic aspects of medical education such as teaching qualifications [24–28] and research training [21]. Our proposed EPA framework adds to this extension of the use of EPAs. The next step would be to complete other sections of the description including required knowledge, skills, attitudes and experiences to allow for summative entrustment, information sources to assess progress and support summative entrustment decisions and supervision level expected at which stage of training for guiding entrustment decisions in HPE PhD training [16, 43].

Furthermore, the EPA framework allows HPE doctoral graduates to appreciate what is being asked of them when they are employed. It could also serve as a guide for the graduates to identify their learning gaps and cultivate their personal development plans. Lastly, the EPAs could offer those aspiring to obtain a doctoral degree with the extent and depth of the expected activities and deepen their understanding of the professional role and also could direct or orient employers who seek- to hire scholars with a doctoral degree in HPE.

## Limitations

This study has limitations. First, it was developed in one country and given the dynamic nature of the field, it may not reflect particular activities expected from HPE doctorate graduates in other settings. Nevertheless, the EPA framework includes basic components that other programs can adapt to their training needs. Second, despite the diversity and broad experience of our expert group, some items relevant to the work of HPE graduates maybe missing in our proposed EPA framework since the concept of EPA is new to HPE doctorate training. Another limitation is that we asked a general question during the brainstorming process, “what do doctor graduates in HPE do after graduation?” to receive more ideas. Although we narrowed the study focus in the subsequent phases, this may have resulted in EPAs beyond the new graduates’ ability. These descriptions should therefore be considered as a framework that can be adjusted over time if needed.

## Conclusions

This study aimed at developing an EPA framework for doctoral graduates in HPE and at obtaining a national consensus in one country. Using NGT followed by a modified Delphi methodology with doctoral graduates in HPE and members of the Board of HPE Examiners, this study led to 24 EPAs, categorized in research and scholarship, educational development and educational management. The proposed EPA framework can be used to improve the HPE doctorate training and to inform employment decisions. A future international consensus procedure could use these EPA outcomes as a starting point. The EPAs also need to be tested in the HPE field to ensure their practical feasibility.

## Supplementary Information

**Additional file 1.**

## Data Availability

All data generated or analysed during this study are included in this published article and its supplementary information files.

## Abbreviations

HPE: Health Professions Education
EPAs: Entrustable Professional Activities
PhD: Doctor of Philosophy
CPD: Continuous Professional Development
