# Supplementary material for: Developing entrustable professional activities for doctoral graduates in health professions education: obtaining a national consensus in Iran
Source: BMC Med Educ. 2022 Jun 2;22:424. doi: 10.1186/s12909-022-03469-6 (PMC9164418; doi:10.1186/s12909-022-03469-6)
Supplement: Supplementary file 1 — Additional file 1. [file 12909_2022_3469_MOESM1_ESM.docx]

Supplemental Table 1. Levels of agreement, means and standard deviations per EPA of the first Delphi round

| **EPAs** | | **EPA title** | **EPA importance** | |
| --- | --- | --- | --- | --- |
|  |  |  | Level of agreement (%) | Mean (SD) |
| **Domain 1: Consultation** | EPA 1 | Providing consultation on research, scholarship and development activities in HPE | 89 | 4.67 (0.71) |
|  | EPA 2 | Providing consultation to faculty members for effective teaching | 100 | 4.67 (0.50) |
|  | EPA 3 | Providing effective feedback to faculty members on teaching and other educational activities | 89 | 4.33 (0.71) |
|  | EPA 4 | Providing consultation on the design, implementation and evaluation of educational programs at various levels | 100 | 4.56 (0.53) |
|  | EPA 5 | Providing advice to educational administrators at different levels of HPE | 100 | 4.33 (0.50) |
|  | EPA 6 | Mentoring faculty members and other stakeholders in HPE | 78 | 4.11 (0.78) |
| **Domain 2: Research & scholarship** | EPA 7 | Performing needs assessment to determine research priorities educational problems | 100 | 5.00 (0.00) |
|  | EPA 8 | Designing research, scholarship and developmental activities in HPE | 78 | 4.33 (0.87) |
|  | EPA 9 | Conducting, collaborating on and directing research, scholarship and developmental activities | 78 | 4.44 (0.88) |
|  | EPA 10 | Contributing in publication of research, scholarship and development activities in HPE | 78 | 4.00 (1.00) |
|  | EPA 11 | Reviewing research and scholarship activities and products in HPE | 78 | 4.11 (1.05) |
| **Domain 3: Education** | EPA 12 | Teaching and facilitating at various educational situations | 100 | 4.56 (0.53) |
|  | EPA 13 | Designing and implementing classes, workshops and other educational events | 89 | 4.67 (0.71) |
|  | EPA 14 | Designing and implementing faculty development programs | 78 | 4.44 (0.88) |
|  | EPA 15 | Designing and implementing educational interventions employing BEME | 100 | 4.78 (0.44) |
|  | EPA 16 | Designing, implementing and revising educational programs | 89 | 4.33 (0.71) |
|  | EPA 17 | Designing and implementing student support systems | 78 | 4.33 (0.87) |
|  | EPA 18 | Designing and producing medical education content | 89 | 4.11 (0.60) |
| **Domain 4: Management** | EPA 19 | Leading and managing change in HPE | 89 | 4.44 (0.73) |
|  | EPA 20 | Formulating Educational policy at different levels of HPE | 78 | 4.22 (0.97) |
|  | EPA 21 | Providing evidence-based solutions in response to various educational problems | 100 | 4.56 (0.53) |
|  | EPA 22 | Developing guidelines and protocols in various areas of HPE | 100 | 3.78 (0.83) |
|  | EPA 23 | Evaluating the cost-effectiveness of educational methods, approaches and services | 78 | 4.00 (0.71) |
| **Domain 5: Evaluation** | EPA 24 | Designing, implementing and evaluating learners' evaluation system at micro to macro levels | 100 | 4.67 (0.50) |
|  | EPA 25 | Designing, implementing and evaluating evaluation systems for the institution and educational program | 100 | 4.78 (0.44) |
|  | EPA 26 | Designing, implementing and evaluating evaluation systems for faculty and educational staff | 78 | 4.33 (0.50) |
|  | EPA 27 | Evaluating the quality of educational products | 78 | 3.78 (0.67) |

HPE stands for Health Profession Education

BEME stands for Best Evidence Medical Education

Supplemental Table 2. Entrustable Professional Activities (titles and descriptions) for Doctoral graduate in Health Profession Education categorized in 'research and scholarship' (pink tables), 'educational development' (blue tables) and 'educational management' (green tables).

| EPA1 Title - Identifying and translating educational needs to research | |
| --- | --- |
| EPA Description | |
| Specifications and Limitations | This EPA describes identifying the problems relevant to the practice of medical education community, investigating knowledge gaps in medical education, formulating research questions, and writing and submitting proposals.  **Specific functions that define this EPA include:**   1. Identifying the problem or knowledge gaps in medical education through:  - personal or professional experiences - communicating with stakeholders (i.e. academics, students, administrators and so forth) - reviewing the literature  1. Formulating a research question or a research goal based on the educational needs and knowledge gaps 2. Developing a proposal including:  - the conceptual framework, description of context, literature review and the study aim - study protocol   - the study team   - an appropriate study design and its details (sampling, data gathering and analyzing methods and procedures, and ethical issues)   - timeline, facilities and cost budgets  1. submitting the proposal for obtaining research finances 2. submitting the proposal to research ethics committees for ethical approval 3. Defending the proposal by responding to reviewers’ comments written or oral   **Limitations:**   1. This EPA also includes educational design research in medical education. 2. This EPA may require external participation and assistance from methodologist or statistician. 3. The purpose of a literature review in this activity is meant to identify the gap and to design a sound research question and study plan. |
| Potential risks in case of failure | Failure to carry out this EPA well can lead to unaddressed educational needs, overlooked research priorities, confusion in the design and conduct of research, unpublishable manuscripts or articles with little impact, unsuccessful educational design research, not receiving research funding, and a waste of resources and costs.  Problems that may occur in implementing this EPA include:   - Failure to identify needs or knowledge gaps, - Generating inappropriate assumptions, - Writing non-standard proposals, - Submitting proposals to unrelated centers or ignoring the recognition of research grants, - Difficulty in obtaining ethical approval, - Ineffective defense from the proposal. |

| EPA2 Title - Conducting and analyzing research | |
| --- | --- |
| EPA Description | |
| Specifications and Limitations | This EPA describes preparing activities for research, conducting research and analyzing the data.  **Specific functions that define this may EPA include:**   1. Providing required resources and facilities 2. Preparing required tools 3. Coordinating with partner centers 4. Sampling and communicating with research samples 5. Collecting information 6. Managing data using appropriate software 7. Analyzing and interpreting findings   **Limitations:**   1. This EPA also includes educational design research in medical education. 2. In this EPA, all types of original studies (quantitative, qualitative and mixed) are implementing. 3. Secondary studies such as systematic reviews, meta-analysis, and realist review and so on are also performing in this activity. 4. This EPA may require external participation and assistance from methodologist or statistician. |
| Potential risks in case of failure | Failure to carry out this EPA may cause unreliable, inconclusive, invalid or irreproducible results, and waste of resources.  Problems that may occur in the implementation of this EPA may include:   - Inability to provide the required resources and facilities, - Failure in the implementation of research protocols, - Ineffective coordination and cooperation, - Sampling error, - Incomplete control of the study, - Insufficient data collection, - Failure in data management, - Bias and error in analyzing and interpreting findings. |

| EPA3 Title - Collaborating, directing and supervising research teams | |
| --- | --- |
| EPA Description | |
| Specifications and Limitations | This EPA describes collaborating, directing and mentoring research teams, and supervising students’ research activities as a member of a research team.  **Specific functions that define this EPA may include:**   1. Supervising students during their research internships 2. Establishing a research team with sufficient expertise and capability 3. Involving students in research activities as a member of a research team 4. Engaging team members in planning and performing research activities 5. Guiding and mentoring the research team members 6. Monitoring the progress of team and its members 7. Improving the working atmosphere and team culture with constructive feedback, open discussions, conflict resolution 8. Evaluating and improving the team performance 9. Collaborating with scientific societies and communities 10. Providing support through grant and award applications, reference letters, training opportunities, personal network connections, etc.   **Limitations: None** |
| Potential risks in case of failure | Failure to carry out this EPA may cause deviation from the research proposal, limit the achievement of the expected results and waste time and resources.  Problems that may occur in implementing this EPA include:   - Lack of knowledge about the abilities of research team member - Ineffective communication and supervision |

| EPA4 Title - Writing, publishing and communicating scientific reports | |
| --- | --- |
| EPA Description | |
| Specifications and Limitations | This EPA describes activities after performing research including preparing and publishing research reports and communicating it with target audience in variety of formats.  **Specific functions that define this EPA may include:**   1. Defining target audience (e.g. scientific community, policy makers, educators, teachers, etc.) 2. Selecting an appropriate communication format (e.g. verbal, written, and visual) and setting (e.g. conference, meeting, journal, etc.) 3. Selecting appropriate content to be communicated, considering target audience and setting 4. Writing and preparing journal articles according to journal’s guidelines containing:    - title and abstract    - problem statement, context and objective(s)    - methodology    - results in the form of texts, tables and figures    - discussion 5. Writing and preparing reports for presentations according to event’s guidelines 6. Determining authorship based on the credible authorship criteria 7. Submitting the report 8. Responding to reviewer comments 9. Presenting reports in the scientific events in various formats including lectures, posters and other items and responding to questions and comments from the audience appropriately   **Limitations:**   1. In this EPA, evidence-based texts such as research guidelines are included, but educational materials and guides are mentioned in another EPA. |
| Potential risks in case of failure | Failure to carry out this EPA may prevent effective communication with other scientific societies, interfere with the writing and publishing the results of educational research and hamper the development of medical education knowledge.  Problems that may occur in implementing this EPA include:   - Failure to follow the criteria and guidelines of journals and scientific societies, - Selection of inappropriate journal, and - Inappropriate interaction with reviewers to make corrections. |

| EPA5 Title - Reviewing research and scholarship activities | |
| --- | --- |
| EPA Description | |
| Specifications and Limitations | This EPA describes reviewing research activities and reports in various formats from journal articles to formal meetings such as defense of proposals and dissertations, seminars and conferences.  **Specific functions that define this EPA may include:**   1. Participating in determining review criteria for research in HPE 2. Reviewing research activities and report critically based on the appropriate criteria 3. Writing a review report and constructive feedback to written reports 4. Criticizing and giving constructive feedback in scientific meetings   **Limitations:**   1. This activity can only be done in an area where the person has expertise and experience. |
| Potential risks in case of failure | Failure to carry out this EPA may reduce the quality of research papers and disrupt the development of medical education researchers.  Problems that may occur in the implementation of this EPA include:   - Failure to employ criteria and standards, subjective review, - Ineffective communication and ineffective feedback providing, - Failure to write an accurate review report |

| EPA6 Title - Consulting on research and scholarship in HPE | |
| --- | --- |
| EPA Description | |
| Specification and Limitations | This EPA describes communicating and consulting to various stakeholders using evidence-based knowledge in relation to planning, conducting and publishing research.  **Specific functions that define this EPA include:**   1. Communicating effectively with the audiences 2. Identifying consultation questions and issues 3. Designing a consultation plan tailored to the audience 4. Gathering the information based on the evidence-based knowledge 5. Conducting an individual or group consultation session and presenting comments and suggestions   **Limitations:**   1. Consulting is limited to issues related to medical education and does not include topics related to psychology or other specialized aspects. 2. Consulting on educational development and management in the field of medical education is done in other EPAs. |
| Potential risks in case of failure | Failure to carry out this EPA may cause the uncovered needs of different stakeholders and ineffective consultation.  Problems that may occur in the implementation of this EPA include:   - lack of effective communication, - lack of use of various means of communication such as social networks and online contact, - incomplete identification of counseling questions, - lack of evidence-based information collection. |

| EPA7 Title - Designing and conducting educational needs assessment | |
| --- | --- |
| EPA Description | |
| Specifications and Limitations | This EPA describes the articulating, elaborating, validating, and prioritizing needs for educational programs and interventions.  **Specific functions that define this EPA may include:**   1. Establishing and leading a needs assessment team or working group 2. Creating a needs assessment plan containing:  - scope and goals - stakeholders and audiences - methods and techniques for sampling and data collection - data analysis methods - prioritization and decision-making methods  1. Communicating the needs assessment plan with related stakeholders 2. Collecting data according to the needs assessment plan 3. Analyzing, interpreting, and prioritizing needs assessment data 4. Translating the defined needs into educational programs and interventions 5. Communicating needs assessment reports in various formats   **Limitations:**   1. The needs assessment in this EPA goes beyond the usual feasibility studies for daily educational activities and interventions. 2. Needs assessment of research activities and identification of the knowledge gap in medical education are covered in another EPA. |
| Potential risks in case of failure | Failure to carry out this EPA well may cause deficient knowledge of important needs of the audiences and stakeholders, actions that are not necessary or desired, and unclear or blind goals for educational programs and interventions.  Problems that may occur in the conduction of this EPA may include:   - Incomplete identification of the audiences and stakeholders, - Developing inappropriate needs assessment plan, - Collection of unnecessary information, - Improper prioritization of needs, - Failure to provide results correctly and to the target audience |

| EPA8 Title - Developing, implementing and revising curricula and educational programs | |
| --- | --- |
| EPA Description | |
| Specifications and Limitations | This EPA describes the transformation of educational needs into curricula and educational programs at different levels of medical education and with the participation of various stakeholders.  **Specific functions that define this EPA may include:**   1. Establishing and leading an educational planning team or working group 2. Developing a curriculum or an educational program containing:  - goals and objectives - content and its organization - educational strategies - teaching methods - student assessment plan and methods - program evaluation plan and methods - communication methods - process management - educational environment improvement - faculty development  1. Communicating the curriculum or educational program with related stakeholders 2. Consulting on implementation and maintenance of the curriculum or educational program 3. Supporting the curriculum or educational program implementation (i.e. faculty development) 4. Revising the curriculum or educational program based on the evaluation results   **Limitations:**   1. Program management is covered in another EPA. 2. The design and implementation of student assessment and program evaluation systems are covered in other EPAs. 3. This EPA requires participation and assistance from experts in educational program subject, and administrators, educational program directors, and other stakeholders. |
| Potential risks in case of failure | Failure to carry out this EPA well may reduce the quality of educational programs and as a result, its goals may not be achieved. It may also disrupt the personal and professional development of learners.  Problems that may occur in implementing this EPA may include:   - Lack of proper curriculum planning model, - Incorrect translation of needs assessment to educational outcomes, - Insufficient stakeholder participation - Inconsistency of program components - Deviation of the program from the set goals - Conflicts between stakeholders, etc. |

| EPA9 Title - Instructional designing for various teaching and learning situations | |
| --- | --- |
| EPA Description | |
| Specifications and Limitations | This EPA describes designing, selecting and implementing learning experiences tailored to the context, and learners’ characteristics and performance using an appropriate model of instructional design.  **Specific functions that define this EPA may include:**   1. Selecting the appropriate model of instructional design 2. Analyzing educational problems and needs, and profile of targeted learners 3. Designing an instructional plan addressing problems and needs, and containing:  - instructional objectives - learning activities - instructional methods - learning assessment blueprint and methods - resources  1. Organizing the actual learning material 2. Implementing instructional design in practice:  - guiding learning activities - using educational materials and media - assessing students' learning - training teacher - eliciting students’ feedback  1. Evaluating the instruction and revising the instructional design   **Limitations:**   1. In this EPA, educational content is only selected and organized, and content production is related to other EPA. |
| Potential risks in case of failure | Failure to carry out this EPA well may cause a lack of a clear and precise instructional design, to reduce instruction effectiveness, and to waste resources.  Problems that may occur in implementing this EPA include:   - Selecting inappropriate instructional design models - Incorrect analysis of situation and audience - Inconsistency of instructional plan with the needs and problems - Improper use of educational materials and media, etc. |

| EPA10 Title - Designing and producing educational content in HPE | |
| --- | --- |
| EPA Description | |
| Specifications and Limitations | This EPA describes designing, organizing and producing educational content in different formats based on the updated knowledge of medical education and presenting it to different audiences.  **Specific functions that define this EPA may include:**   1. Analyzing the situation and audiences 2. Searching, selecting and validating educational resources 3. Selecting or designing templates, media and materials 4. Producing educational content in various formats, including textbooks, learning guides, slides, video clips, multimedia software, etc. 5. Collaborating with an educational content production team 6. Evaluating the quality of the educational content   **Limitations:**   1. This EPA may require participation and assistance from experts in educational technology and other fields. |
| Potential risks in case of failure | Failure to carry out this EPA well may reduce the quality of educational content and its usefulness and attractiveness for the audience.  Problems that may occur in the implementation of this EPA include:   - The content is not relevant to the audience and situation, - Use of unreliable resources for producing content, - Incompatibility of format and media with content. |

| EPA11 Title - Teaching and facilitating in various educational situations | |
| --- | --- |
| EPA Description | |
| Specifications and Limitations | This EPA describes teaching practice as well as facilitating group learning activities for audiences at different levels.  **Specific functions that define this EPA may include:**   1. preparing a course plan or lesson plan by determining:    - course details and learners’ characteristics    - general and specific objectives    - course schedule    - educational strategies    - teaching methods    - learning activities    - learning assessment 2. Providing teaching using different and appropriate methods and techniques 3. employing materials and various media in teaching to enhance learning 4. Facilitating group learning using facilitation techniques 5. Managing teaching sessions and learning processes 6. Evaluating the quality and effectiveness of learning processes   **Limitations:**   1. Academic presentations and management of scientific meetings and events are mentioned in other EPAs. |
| Potential risks in case of failure | Failure to carry out this EPA disrupts the training of students and the development of faculty members and other stakeholders.  Problems that may occur in the implementation of this EPA include:   - defect in the lesson plan, - insufficient knowledge of the characteristics of the audience, - Inappropriate use of teaching methods and techniques, - Failure to facilitate learning groups, - Problems in learning assessment, for example in designing tests |

| EPA12 Title - Mentoring stakeholder groups in HPE | |
| --- | --- |
| EPA Description | |
| Specifications and Limitations | This EPA describes mentoring activities that are provided for HPE stakeholders groups including students, faculty members and peers (mentees) to support the academic, professional and personal aspects of their careers.  **Specific functions that define this EPA may include:**   1. Communicating with stakeholder groups in different ways, face to face or remotely 2. Analyzing the needs of the stakeholder groups 3. Planning and adjusting the mentoring and support program according to the identified needs of the special group of stakeholders 4. Providing mentoring services including personal, academic and professional support 5. Evaluating the extent of goal achievement and performance improvement   **Limitations:**   1. Financial and organizational support is not provided in this EPA. |
| Potential risks in case of failure | Failure to carry out this EPA may disrupt the personal, academic and professional support of students, faculty members, peers and other stakeholders in HPE.  Problems that may occur in the implementation of this EPA include:   - Ineffective communication with the stakeholder groups, - Failure to identify needs and provide unnecessary support services, - Lack of a structured and scheduled plan, - Failure to evaluate the results of the support |

| EPA13 Title - Reviewing educational materials and products | |
| --- | --- |
| EPA Description | |
| Specifications and Limitations | This EPA describes reviewing educational products and materials according to the quality standards.  **Specific functions that define this EPA may include:**   1. Setting the quality standards for educational products and materials 2. Reviewing educational products and materials based on the predetermined standards 3. Communicating and providing effective and constructive feedback to the audiences 4. Reporting the review results in suitable formats   **Limitations:**   1. In this EPA, educational products and materials such as curricula, books, pamphlets, multimedia, videos are among the items to be reviewed. 2. Reviewing research proposals and reports, dissertations or scientific papers are introduced in other EPA. 3. Evaluating educational programs is carried out in a separate EPA. |
| Potential risks in case of failure | Failure to carry out this EPA may cause the use of useless educational products and materials and waste of resources  Problems that may occur in the implementation of this EPA include:   - Setting inappropriate standards, - Bias in review, - Ineffective feedback |

| EPA14 Title - Designing, implementing and revising student assessment system | |
| --- | --- |
| EPA Description | |
| Specifications and Limitations | This EPA describes designing, implementing and revising student assessment systems to ensure that students acquired competencies.  **Specific functions that define this EPA may include:**   1. designing the framework of the student assessment system containing:  - purpose of the assessment - rules and regulations - domain mapping (content, dynamic tools, etc.) - assessment schedule - assessors - assessment context and setting - valuing information (scoring and standard setting, etc.) - infrastructure  1. Supporting the student assessment system (i.e. faculty development) 2. Participating in collecting, combining, valuing, interpreting and reporting information 3. Monitoring, evaluating, improving, and revising the student assessment system   **Limitations:**   1. This EPA requires external participation and assistance from administrators, faculty members, staff and other stakeholders. |
| Potential risks in case of failure | Failure to carry out this EPA may lead to incompetent graduates, unsystematic student assessment and waste of resources.  Problems that may occur in the implementation of this EPA include:   - Lack of a comprehensive framework for student assessment. |

| EPA15 Title - Designing, implementing and revising the faculty evaluation system | |
| --- | --- |
| EPA Description | |
| Specifications and Limitations | This EPA describes designing, implementing and revising the faculty evaluation system with the aim of improving the faculty members' competencies and promoting the accountability of the educational system.  **Specific functions that define this EPA may include:**   1. Designing the framework of the faculty evaluation system containing:  - purpose of the faculty evaluation - evaluation domains, criteria and standards - sources, methods and tools of collecting information - procedures for collecting information - valuing information (scoring and standard setting, etc.) - infrastructure  1. Participating in collecting, combining, valuing, interpreting and reporting information 2. Monitoring, evaluating, improving, and revising the faculty evaluation system   **Limitations:**   1. This EPA requires external participation and assistance from administrators, faculty members, staff and other stakeholders. |
| Potential risks in case of failure | Failure to carry out this EPA may result in low-quality education.  Problems that may occur in the implementation of this EPA include:   - Lack of a comprehensive framework for faculty evaluation. |

| EPA16 Title - Designing, implementing and revising quality assurance system | |
| --- | --- |
| EPA Description | |
| Specifications and Limitations | This EPA describes designing, implementing and revising the quality assurance system of educational programs and institutions to monitor and improve processes and performance.  **Specific functions that define this EPA may include:**   1. Designing the framework of the quality assurance system containing:  - quality assurance model - evaluation objectives, criteria and standards - sources, methods and tools of collecting information - procedures for collecting information - infrastructure  1. Participating in collecting, combining, valuing, interpreting and reporting information 2. Monitoring, evaluating, improving, and revising the quality assurance system   **Limitations:**   1. This EPA requires external participation and assistance from administrators, faculty members, staff and other stakeholders. |
| Potential risks in case of failure | Failure to carry out this EPA may result in low-quality education.  Problems that may occur in the implementation of this EPA include:   - Lack of a comprehensive framework for quality assurance. |

| EPA17 Title - Consulting on planning, teaching and learning processes, and evaluation activities | |
| --- | --- |
| EPA Description | |
| Specifications and Limitations | This EPA describes communicating and consulting to various stakeholders using evidence-based knowledge in relation to educational planning, teaching and learning processes and evaluation activities.  **Specific functions that define this EPA may include:**   1. Communicating effectively with the audiences 2. Identifying consultation questions and issues 3. Designing a consultation plan tailored to the audience 4. Gathering the information based on the evidence-based knowledge 5. Conducting an individual or group consultation session and presenting comments and suggestions   **Limitations:**   1. Consulting is limited to issues related to medical education and does not include topics related to psychology or other specialized aspects. 2. Consulting on research and management in the field of medical education is done in other EPAs. |
| Potential risks in case of failure | Failure to carry out this EPA may cause the uncovered needs of different stakeholders and ineffective consultation.  Problems that may occur in the implementation of this EPA include:   - Lack of effective communication, - Lack of use of various means of communication such as social networks and online contact, - Incomplete identification of counseling questions, - Lack of evidence-based information collection. |

| EPA18 Title - Analyzing, formulating and revising educational policies | |
| --- | --- |
| EPA Description | |
| Specifications and Limitations | This EPA describes analyzing and revising current educational practices and policies, and formulating new educational policies with the participation of administrators and other stakeholder groups.  **Specific functions that define this EPA may include:**   1. Identifying educational problems and issues through different sources 2. Selecting educational issues and setting a policy agenda 3. Communicating with stakeholder groups and engaging them in the policy cycle. 4. Formulating, analyzing, and comparing policy options 5. Consulting on a decision-making process for the selection of policy options 6. Participating in educational policy implementation 7. Monitoring, evaluating and revising educational policies   **Limitations:**   1. This activity is often done in partnership with administrators and related centers and departments. |
| Potential risks in case of failure | Failure to carry out this EPA may lead to misdirection of the education system at different levels and disrupt the coordination of its components, resulting in reduced effectiveness and waste of resources and costs.  Problems that may occur in the implementation of this EPA include:   - ineffective communication with stakeholders, - difficulties in identifying priorities, - failure to identify and select policies, - lack of a standard policy plan - lack of cooperation and guidance of policy working groups. |

| EPA19 Title - Designing, implementing and evaluating reforms | |
| --- | --- |
| EPA Description | |
| Specification and Limitations | This EPA describes analyzing current issues and problems in various components of the educational system, designing and implementing reforms, and evaluating its effectiveness.  **Specific functions that define this EPA may include:**   1. Assessing the educational needs and creating a sense of urgency for reform by:    - identifying existing strengths, weaknesses, threats and opportunities    - discussing with stakeholder groups the identified needs    - seek support from stakeholder groups 2. Assembling a competent team with shared understanding of purposes and responsibilities 3. selecting and prioritizing reform alternatives 4. participating in the implementation of reform 5. Empowering involved groups in reform 6. Communicating reform process and results with stakeholder groups 7. Monitoring and evaluating the reform process and results   **Limitations:**   1. This activity is often done in partnership with managers and related centers and departments. |
| Potential risks in case of failure | Failure to carry out this EPA may cause ignoring problems and failure to prevent serious challenges facing the education system.  Problems that may occur in the implementation of this EPA include:   - Failure to identify problems and needs, - Lack of planning for reform, - Failure to communicate and align stakeholders. |

| EPA20 Title - Designing, implementing and evaluating personal and professional support and development programs | |
| --- | --- |
| EPA Description | |
| Specifications and Limitations | This EPA describes designing, implementing and evaluating programs to support students, faculty members, administrators, etc. in personal and professional development.  **Specific functions that define this EPA may include:**   1. Analyzing academic environment and determining the components of the personal and professional support system 2. Designing a personal and professional support system tailored to the needs of the audience 3. Developing empowerment program for support system implementers, 4. Evaluating the quality and effectiveness of the personal and professional support system   **Limitations:** None |
| Potential risks in case of failure | Failure to carry out this EPA may cause the ignorance of the personal and professional needs of stakeholders.  Problems that may occur in the implementation of this EPA include:   - Failure in academic analysis and need assessment and - Designing ineffective support system. |

| EPA21 Title - Managing organizational processes and resources | |
| --- | --- |
| EPA Description | |
| Specifications and Limitations | This EPA describes managing organizational processes and resources at different hierarchical levels of health professions education institutions.  **Specific functions that define this EPA may include:**   1. Translating institutional policies into specific strategies and action plans 2. Organizing and allocating the facilities, human resources and budget 3. Supervising, guiding and directing employees in performing planned activities 4. Coordinating, monitoring and supervising various organizational processes   **Limitations:** None |
| Potential risks in case of failure | Failure to implement this EPA may result in waste of resources and incoherency and inefficiency in the activities of educational or research units under guidance.  Problems that may occur in the implementation of this EPA include:   - lack of identification of all available resources and facilities - inability to allocate them properly, - ineffective communication in the workplace - inability to direct and harmonize functions and insufficient monitoring. |

| EPA22 Title - Managing and supervising projects | |
| --- | --- |
| EPA Description | |
| Specifications and Limitations | This EPA describes managing educational projects in terms of planning, executing and following up.  **Specific functions that define this EPA may include:**   1. Developing the project plan containing:  - goals and scope - risk analysis - resources and budget - task and relationship analysis - timeline - evaluation methods  1. Assembling a competent team with shared understanding of purposes and responsibilities 2. Executing the project and assign tasks to the employees or team members that fit them 3. Designing and implementing knowledge management system 4. Monitoring and controlling the project in terms of:  - project objectives - quality deliverables - effort and cost tracking - project performance  1. Evaluating and finalizing the project   **Limitations:** None |
| Potential risks in case of failure | Failure to carry out this EPA may cause problems in executing projects and not achieving the expected goals.  Problems that may occur in the implementation of this EPA include:   - Lack of comprehensive plan, - Problems in assembling the team, - Lack of knowledge on available resources, - Failure to monitor performance, - Disregard for the knowledge management system and lessons learned. |

| EPA23 Title - Analyzing the cost-effectiveness of practices and interventions | |
| --- | --- |
| EPA Description | |
| Specifications and Limitations | This EPA describes analyzing the cost-effectiveness of various medical education practices and approaches to decide on maintaining or discarding them.  **Specific functions that define this EPA include:**   1. Defining the scope of analysis and determining the components and details of interventions 2. Choosing the perspective and type of cost-effectiveness analysis 3. Designing the cost-effectiveness analysis by:  - Identifying effectiveness indicators - Identifying and valuing costs  1. Collecting and recording data from available sources 2. Calculating the cost effectiveness 3. reporting the results and interpretation to various stakeholders   **Limitations:** None |
| Potential risks in case of failure | Failure to carry out this EPA may lead to the continuation of approaches and activities that do not have the necessary effectiveness while incurring exorbitant costs.  Problems that may occur in the implementation of this EPA include:   - Lack of proper model and analysis plan, - Insufficient information, - Computational error, - Lack of familiarity with software, - Bias in analysis and conclusion, - Lack of accurate report writing. |

| EPA24 Title - Consulting on management and leadership | |
| --- | --- |
| EPA Description | |
| Specifications and Limitations | This EPA describes communicating and consulting to various stakeholders using evidence-based knowledge in relation to management and leadership.  **Specific functions that define this EPA include:**   1. Communicating effectively with the audiences 2. Identifying consultation questions and issues 3. Designing a consultation plan tailored to the audience 4. Gathering the information based on the evidence-based knowledge 5. Conducting an individual or group consultation session and presenting findings and suggestions   **Limitations:**   1. Consulting on educational development and research in the field of medical education is done in other EPAs. |
| Potential risks in case of failure | Failure to carry out this EPA may cause the uncovered needs of different stakeholders and ineffective consultation.  Problems that may occur in the implementation of this EPA include:   - Lack of effective communication, - Lack of use of various means of communication such as social networks and online contact, - Incomplete identification of counseling questions, - Lack of evidence-based information collection. |
